# Bocavirus Infection in Otherwise Healthy Children with Respiratory Disease

**DOI:** 10.1371/journal.pone.0135640

**Published:** 2015-08-12

**Authors:** Nicola Principi, Antonio Piralla, Alberto Zampiero, Sonia Bianchini, Giulia Umbrello, Alessia Scala, Samantha Bosis, Emilio Fossali, Fausto Baldanti, Susanna Esposito

**Affiliations:** 1 Pediatric Highly Intensive Care Unit, Department of Pathophysiology and Transplantation, Università degli Studi di Milano, Fondazione IRCCS Ca’ Granda Ospedale Maggiore Policlinico, Milan, Italy; 2 Molecular Virology Unit, Microbiology and Virology Department, Fondazione IRCCS Policlinico San Matteo, Pavia, Italy; 3 Emergency Unit, Fondazione IRCCS Ca’ Granda Ospedale Maggiore Policlinico, Milan, Italy; 4 Section of Microbiology, Department of Clinical, Surgical, Diagnostic and Pediatric Sciences, University of Pavia, Pavia, Italy; Kliniken der Stadt Köln gGmbH, GERMANY

## Abstract

To evaluate the role of human bocavirus (hBoV) as a causative agent of respiratory disease, the importance of the viral load in respiratory disease type and severity and the pathogenicity of the different hBoV species, we studied all hBoV-positive nasopharyngeal samples collected from children who attended an emergency room for a respiratory tract infection during three winters (2009–2010, 2011–2012, and 2013–2014). Human bocavirus was detected using the respiratory virus panel fast assay and real-time PCR. Of the 1,823 nasopharyngeal samples, 104 (5.7%) were positive for hBoV; a similar prevalence was observed in all three periods studied. Among hBoV-infected children, 53.8% were between 1–2 years old, and hBoV was detected alone in 57/104 (54.8%) cases. All of the detected hBoV strains belonged to genotype 1. The median hBoV load was significantly higher in samples containing strains with both the N546H and T590S mutations compared to other samples (p<0.05). Children with a single hBoV-1 infection more frequently had upper respiratory tract infections (URTIs) than those who were co-infected (37.0% vs 17.8%, respectively, p = 0.04). The duration of hospitalization was longer among children with high viral loads than that observed among children with low viral loads (8.0 ±2.2 days vs 5.0 ±1.5 days, respectively, p = 0.03), and the use of aerosol therapy was more frequent among children with high viral loads than among those with low viral loads (77.1% vs 55.7%, respectively, p = 0.04). This study shows that hBoV is a relatively uncommon but stable infectious agent in children and that hBoV1 seems to be the only strain detected in Italy in respiratory samples. From a clinical point of view, hBoV1 seems to have in the majority of healthy children relatively low clinical relevance. Moreover, the viral load influences only the duration of hospitalization and the use of aerosol therapy without any association with the site of the respiratory disease.

## Introduction

Human bocavirus (hBoV) is a recently identified viral agent that belongs to the family *Parvoviridae* and contains a single linear positive-sense or negative-sense single-stranded deoxyribonucleic acid genome [1]. This virus has been detected mainly in younger children, in nasopharyngeal secretions, in sera and blood samples of patients with upper (URTI) and lower (LRTI) respiratory tract infections and in faecal specimens of subjects with gastroenteritis [2]. Currently, hBoVs are classified into species 1 through 4; hBoV1 is predominantly found in the respiratory tract, and hBoV2, hBoV3, and hBoV4 are found mainly in stool [3].

Despite there are studies suggesting that hBoV is able to infect the lower airways causing severe infections in both children and adults, the role of hBoV as a causative agent of respiratory disease is frequently questioned due to its common detection with other potential pathogens [4] and the evidence that in some studies co-infections can have a significantly greater clinical and socioeconomic impact on infected children and their households than hBoV infection alone [5]. Moreover, the importance of the viral load in determining the type and severity of respiratory disease as well as the pathogenicity of the different hBoV species [6] are not precisely defined. The main aim of this study was to contribute to resolving these problems. The circulation of hBoV during several winter seasons in Italy was investigated, and a phylogenetic analysis of detected strains was performed. In addition, correlations between different hBoV strains and the severity of disease in cases with infections due to hBoV alone or due to co-infections were studied. Finally, the role of the viral load was analysed.

## Methods

### Study design

To evaluate the circulation of the different hBoV types and the possible relationships between viral load, virus genetic characteristics, and the severity of infection, nasopharyngeal swabs were collected from otherwise healthy children attending the emergency room of the Fondazione IRCCS Ca’ Granda Ospedale Maggiore Policlinico, University of Milan, Italy, due to a respiratory tract infection arising between November 1 and March 31 during 3 winters (2009–2010, 2011–2012, and 2013–2014). The study was approved by the Ethics Committee of the Fondazione IRCCS Ca’ Granda Ospedale Maggiore Policlinico, Milan, Italy. Written informed consent of a parent or legal guardian was required, and children ≥ 8 years of age were asked to give their written assent. Patients’ demographic characteristics and medical histories were retrieved from hospital charts and were systematically recorded before and after the first visit to the emergency room using standardized written questionnaires [7]. The study patients were classified into disease groups (i.e., acute otitis media, rhinosinusitis, pharyngitis, croup, infectious wheezing, acute bronchitis, pneumonia) on the basis of signs and/or symptoms using well-established criteria and were finally subdivided into two subgroups: upper (URTIs) and lower respiratory tract infections (LRTIs) [8]. Nasopharyngeal secretions were collected from all of the children immediately after admission to the emergency room using a paranasal flocked swab (1 swab per child), which was stored in a tube containing 1 mL of universal transport medium (Kit Cat. No. 360c, Copan Italia, Brescia, Italy).

### Respiratory virus identification

Viral nucleic acids were extracted from each swab by means of a Nuclisens EasyMAG automated extraction system (Biomeriéux, Craponne, France), and the extract was tested for respiratory viruses using the respiratory virus panel xTAG RVP FAST v2 (Luminex Molecular Diagnostics, Inc., Toronto, Canada), which simultaneously detects influenza A virus (subtypes H1 or H3); influenza B virus; respiratory syncytial virus (RSV) types A and B; human parainfluenza virus types 1–4 (hPiV1-4); adenovirus (AdV); human metapneumovirus (hMPV); coronaviruses (hCoV) 229E, NL63, OC43 and HKU1, enterovirus/rhinovirus (EV/hRV); and hBoV, in accordance with the manufacturer’s instructions [9, 10]. Samples that were positive for hBoV were stored at -80°C.

### HBoV real-time polymerase chain reaction (PCR)

Viral nucleic acid extracts previously testing positive for hBoV were re-tested for confirmation by two different singleplex real-time PCRs using TaqMan Universal Master Mix II (Applied Biosystems, California, USA). Amplification and detection of viral DNA were performed with a 7900HT real-time PCR System machine (Applied Biosystems, California, USA). Conserved regions for RT-PCR primers and probes were identified in the hBoV NS-1 and NP-1 genes from the nucleotide sequence alignments available from GenBank (for NS1, DQ206700-08, DQ000495-96, and DQ200648, and for NP-1, DQ000495-96, AB243566-72, DQ296618-35, DQ353695-99, DQ299885, DQ267760-75, DQ284856, DQ295844, and AM109958-66; http://www.ncbi.nlm.nih.gov/genbank/). Each 25 μL singleplex reaction mixture consisted of 0.5 μL of forward primer 5’-TGCAGACAACGCYTAGTTGTTT-3’ and reverse primer 5’-CTGTCCCGCCCAAGATACA-3’ for the 88 base pair NS-1 target or forward primer 5’-AGCATCGCTCCTACAAAAGAAAAG-3’ and reverse primer 5’-TCTTCATCACTTGGTCTGAGGTCT-3’ for the NP-1 target, 0.125 μL of probe 5’-CCAGGATTGGGTGGAACCTGCAAA-3’ or 5’-AGGCTCGGGCTCATATCATCAGGAACA-3’, and 2.5 μL of sample viral DNA. PCRs were conducted at 50°C for 2 min and then at 95°C for 10 min, followed by 45 cycles at 95°C for 15 sec and at 60°C for 1 min. TaqMan probes were labelled at the 5’ ends with the reporter molecule 6-carboxyfluorescein and at the 3’ ends with Black Hole Quencher 1 (Biosearch Technologies, Inc., Novato, CA). Each run included one synthetic template control and one no-template control for each target. Specimen extracts were also tested by real-time PCR for the human RNase-P gene to monitor specimen quality and the presence of PCR inhibitors. A positive test for both the NS1 and NP-1 targets or for a single target confirmed from a second extraction from a new sample aliquot was considered definitive evidence of hBoV infection.

The viral load was obtained using real-time PCR with the NS1 primers and probe previously described and a DNA plasmid used as the standard calibrator. The amplified target fragment of the plasmid was verified by sequencing. Plasmid DNA concentrations were detected with an ND-1000 spectrophotometer (NanoDrop products, Wilmington, DE, USA). Each run included plasmid and negative controls. Standard precautions were taken throughout the PCR process to avoid cross-contamination. Negative controls and serial dilutions of the positive controls were included in every PCR assay. Finally, quantitative results were reported as DNA copies/mL of respiratory samples. The viral load was defined as low for values ≤10^6^ log (copies/mL) and as high for values >10^6^ log (copies/mL).

### HBoV sequencing

For genotyping, the viral VP-1/2 gene was amplified using a conventional PCR assay. Briefly, 4 sets of forward and reverse primers (5’-CACAGACAGAAGCAGACGAGAT-3’ and 5’-GGTGAGAAGTGACAGCTGTATTG-3’; 5’-TTCAGAATGGTCACCTCTACA-3’ and 5’-CTGTGCTTCCGTTTTGTCTTA-3’; 5’-AACTTTGACTGTGAATGGGTTA-3’ and 5’-AAATAGTGCCTGGAGGATGAT-3’; 5’-CTATCACCAGAGAAAATCCAATC-3’ and 5’-GAGACGGTAACACCACTA-3’) were used in PCR amplification and the Quantitect Probe Master Mix (QIAGEN, Venlo, Netherlands) was used as the basis for the reaction mix. Viral products were analysed by electrophoresis on a 1.5% agarose gel and purified with the QIAquick Gel Extraction Kit (QIAGEN, Venlo, Netherlands). Sequencing reactions were set up with purified DNA, one of the specific primers used in the PCR and BigDye Terminator v3.1 Cycle Sequencing Kit (Applied Biosystems, California, USA) according to the protocol recommended by the manufacturer. Sequencing and sequence analysis were performed on a 3130 Genetic Analyser (Applied Biosystems, California, USA).

### Sequence analysis

All alignments were performed using ClustalX 2.1 and BioEdit (version 7.1.3.0) software (Ibis Biosciences, Carlsbad, CA). Phylogenetic trees of the VP-1/2 protein gene were generated using the neighbour-joining method and p-distance model of the Molecular Evolutionary Genetics Analyses (MEGA) software, version 5.05 [11]. Bootstrap probabilities for 1,000 iterations were calculated to evaluate confidence estimates. The graphs were made using GraphPad Prism version 5.01 for Windows (GraphPAD Software, San Diego, CA). All genotyped sequences of the hBoV VP-1/2 gene were submitted to GenBank (accession numbers KR014412-KR014516).

### Selective pressure analyses

Tests for positive selection were conducted on the Datamonkey server [12] using the single-likelihood ancestor (SLAC), and the fixed-effects likelihood (FEL) [13], the internal branch fixed-effects likelihood (IFEL) [14], the mixed effects model of evolution (MEME) [15], and fast unconstrained Bayesian approximation methods (FUBAR). The dN/dS ratios were calculated using the SLAC and FEL codon-based maximum likelihood approaches. The SLAC approach counts the number of non-synonymous changes per non-synonymous site (dN) and tests whether it is significantly different from the number of synonymous changes per synonymous site (dS). The FEL approach estimates the ratios of non-synonymous to synonymous changes for each site in an alignment. The IFEL method is similar to the FEL, but tests site-by-site selection along only the internal branches of the phylogeny. In order to avoid an excessive false-positive rate, sites with SLAC, FEL, IFEL and MEME p-values of <0.1 and a FUBAR posterior probability of >0.90 were accepted as candidates for selection.

### Statistical analysis

Descriptive statistics of the responses were generated. Continuous variables were presented as mean values and standard deviations (SDs) and categorical variables as numbers and percentages. For categorical data, comparisons between groups were performed using a contingency table analysis with the *Χ* ^2^ or Fisher’s exact test when appropriate. For ordered categorical data, a Cochran-Armitage test for trend was used to compare the groups. Continuous data were analysed using a two-sided Student’s t-test after ensuring the data were normally distributed (based on the Shapiro-Wilk statistic) or using a two-sided Wilcoxon’s rank-sum test if the data were non-normal. All analyses were two tailed, and p-values of 0.05 or less were considered to be statistically significant. All analyses were conducted using SAS version 9.2 (Cary, NC, USA).

## Results

### HBoV incidence and genotypes

During the three study periods, 1,823 nasopharyngeal samples were collected in the emergency room. Of these, 104 (5.7%) tested positive for hBoV (Table 1). Among hBoV infected children, 53.8% were between 1–2 years old, whereas 28.8% and 17.3% were aged <1 and ≥3 years, respectively. The prevalence of hBoV detection was quite similar in the three studied periods; 30 (28.6%), 39 (37.1%), and 36 (34.3%) positive samples were collected in the winter seasons of 2009–2010, 2011–2012, and 2013–2014, respectively. HBoV was the only virus detected in 57/104 (54.8%) cases and was detected in association with one (89.5%) or more (10.5%) viruses in 47 (45.2%) cases. EV/hRV and RSV were the most common co-infecting viral agents and were found, respectively, in 20 and 18 samples. Subjects with co-infection were younger than those without (p = 0.03). Considering 10^6^ DNA copies/mL as a cut-off, the viral load was classified as low in 66 (63.5%) cases and as high in 38 (36.5%) cases.

**Table 1:** Positivity for bocavirus in children with respiratory tract infections, according to age group and presence of co-infection(s).

	No. (%) of positive samples				
Age group (years)	Overall	Without co-infection	With co-infection(s)	Low viral load	High viral load
< 1	30 (28.8)	12 (21.0)	18 (38.3)	22 (33.3)	8 (21.0)
1–2	56 (53.8)	32 (56.1)	24 (51.1)	31 (47.0)	25 (65.8)
≥ 3	18 (17.3)	13 (22.8)	5 (10.6)	13 (19.7)	5 (13.2)
**Total**	104	57	47	66	38

One missing value for age.

Viral load was categorized in two groups, and was considered “low” for values ≤6 log (copies/mL) and “high” for values >6 log (copies/mL).

p-value = 0.03 for comparison between subgroups of presence of co-infection(s), according to age group (Cochran-Armitage trend test).

p-value = 0.67 for comparison between subgroups of viral load, according to age group (Cochran-Armitage trend test).

### Phylogenetic analyses and amino acid signatures

**Fig 1:**
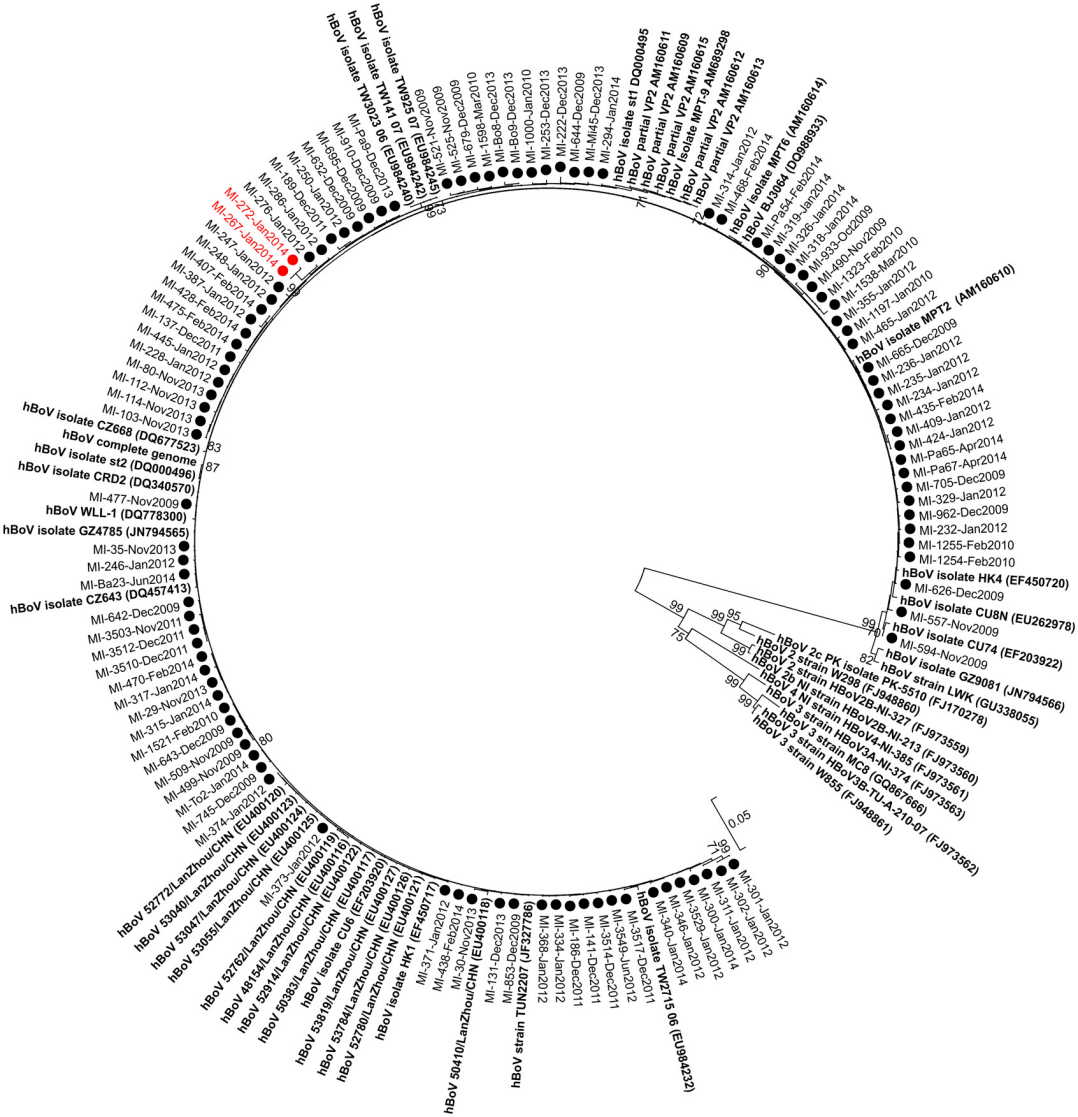
Phylogenetic tree based on complete VP1/VP2 gene sequences of human bocavirus (hBoV) strains. Sequences originating from this study are indicated with black and red circles. HBoV reference stains are in bold. The percentage of replicate trees in which the associated taxa clustered together in the bootstrap test (1,000 replicates) are shown next to the branches.

The phylogenetic tree constructed using the VP1/VP2 sequences showed that all of the Italian hBoV strains detected during the three study periods belonged to hBoV genotype 1 (Fig 1). No unusual clustering was observed among the identified strains; hBoVs circulating in 2009 were closely related to strains circulating in 2014. The sequence identity matrix of the VP1/VP2 gene showed minimum to maximum identity ranges of 97.8–100% between the Italian strains and 98.4–99.7% with respect to hBoV st1 reference strains (DQ000495). In comparison to the reference strain, 8/105 (7.6%) strains had only one amino acid difference, 32/105 (30.4%) strains had two amino acid differences, and the remaining strains (65/105; 61.9%) had at least three amino acid changes.

**Fig 2:**
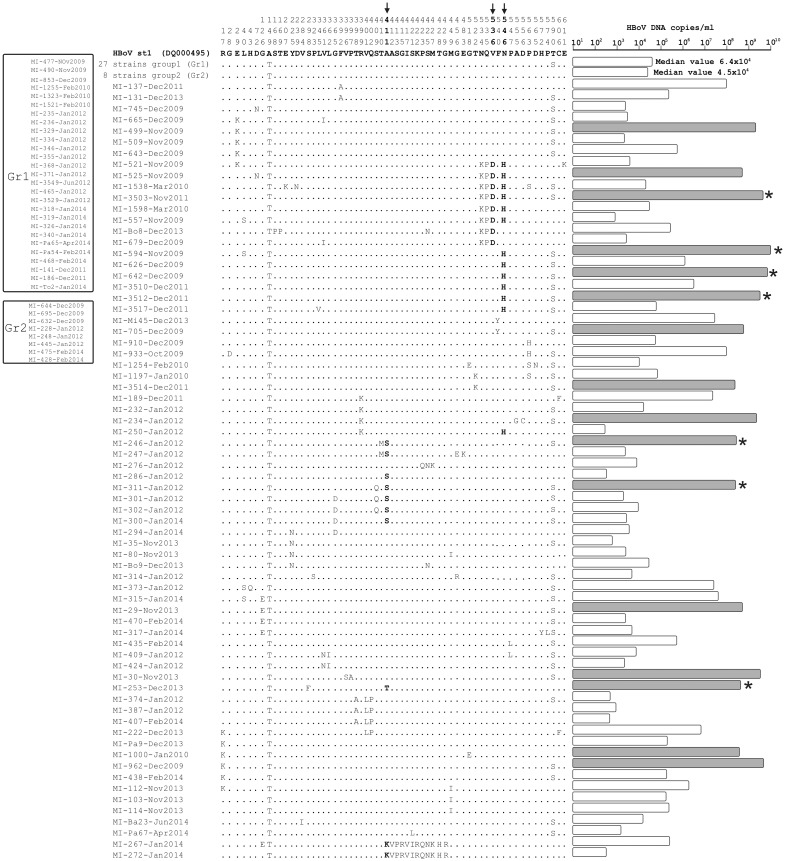
Alignment of VP1/VP2 amino acid changes with respect to the prototype strain (human bocavirus [hBoV] st1; DQ000495) in 105 Italian hBoV strains. Dots indicate identity. Amino acids highlighted in bold are under positive selective pressure. The bar length corresponds to the viral load levels. The viral load levels of strains with the T590S change and an additional change in a codon under positive selection are marked with an asterisk.

A total of 61/672 (9.1%) amino acid positions were observed to have at least one change in the VP1/VP2 sequence alignments (Fig 2). Of these, 7/61 (11.5%) changes occurred within the VP1 unique (VP1U) region corresponding to the first 129 amino acids at the N-terminus of the VP1 gene. Specifically, the following changes were observed: R17K, G28D, E29K, L40S, H43Q, D72N, and G126E (Fig 2).

The VP1U region includes the conserved phospholipase A_2_ (PLA_2_) motif (nt 21–63). The VP1U sequences of all hBoV isolates identified in this study revealed conserved YXGXG (nt 16–20) and HDXXY (nt 41–45) motifs in the catalytic site of secreted PLA_2_. In addition, the amino acid residues at positions 21, 41, 42 and 63 have been hypothesized to form the catalytic network for enzymatic activity. In our hBoV strains, all the sequences had amino acids associated with efficient enzymatic activity (P21, H41, D42, and D63).

Of note, two hBoV strains (MI-267-Jan2014 and MI-272-Jan2014) had a peculiar amino acid sequence in the 19-amino acid segment starting at amino acid 411 (KVPTRRVQPYIRQTNWKHR), which has not been previously reported in hBoV strains included in the GenBank database (in red, Fig 1). Overall, these two strains had 13 and 14 amino acid changes compared to the hBoV st1 strain, and almost all these changes were included in the region described below. The origin of this highly divergent region, which occurred in spite of the conservation displayed in the rest of the hBoV DNA genome, remains to be defined.

### Selective pressure

A global analysis of selective pressure made using the SLAC model indicated an estimated overall dN/dS ratio of 0.18. Overall, the site-specific analyses identified three sites (411, 536, and 546) as under positive selection by at least two methods used (SLAC, FEL, FUBAR, and MEME). The IFEL model was used to determine the selection pressure acting on the VP1/VP2 codons along the internal branches of the tree. Two positively selected codons (411 and 546) were identified. The selected sites, highlighted with arrows in Fig 2, were presumably located in the external loop of the VP1 or VP2 protein. In detail, the Ala (A) to Ser (S) mutation was observed at codon 411 and occurred in 7 hBoV strains circulating in January 2012, the Ala (A) to Thr (T) mutation was observed in one strain circulating in December 2013, and the Ala (A) to Lys (K) mutation was observed in two strains circulating in January 2014. The Val (V) to Asp (D) mutation at codon 536 was observed in 8 hBoV strains circulating in the 2009–11 and 2013 seasons which were associated with N534K and Q535P mutations. Finally, the Asn (N) to His (H) mutation at codon 546 occurred in 13 hBoV strains circulating in the 2009–2012 seasons. Of these strains, 6/13 (46.1%) were also characterized by the N534K, Q535P, and Q535D mutations. Several negatively selected sites were identified by different methods (Table 2).

**Table 2:** Positively and negatively selected sites for human bocavirus strains originating from this study.

Method	Positive	Negative
SLAC	None	29 sites
FEL	**536**, **546**	34 sites
FUBAR	17, 40, 225, 361, **411**, 421, 534, 535, **536**, **546**, 563	38 sites
IFEL	**411**, **546**	17 sites
MEME	225, 361, 392, 396, 401, 409, 410, **411**, 413, 421, 428, **546**, 547, 556, 563	-

FEL: fixed-effects likelihood; FUBAR: fast unconstrained Bayesian approximation methods; IFEL: internal branch fixed-effects likelihood; MEME: mixed effects model of evolution; SLAC: single-likelihood ancestor.

### Mutations and viral load

Regarding the viral load, a wide range of hBoV DNA levels from 3.5x10^2^ to 7.5x10^9^ copies/mL were found in the clinical samples. In Fig 2 (right side), the viral load of each Italian hBoV strain is reported near the aligned mutations. In the group of strains (n = 70; 66.6%) harbouring at least 2 mutations in addition to A149T, the values of the hBoV load greater than 1x10^8^ DNA copies/ml are reported in grey. A total of 16 strains had a very high viral load, and 13/16 (81.3%) harboured the T590S mutation. This percentage is nearly significantly different than the overall frequency (42/70; 53.8%) of T590S in the group of strains described below (p = 0.08). Seven of the 13 strains with the T590S mutation had an additional mutation in one of the sites under positive selection (reported in Fig 2 with an asterisk). In detail, 4/13 (30.8%) had the N546H change, 2/13 (15.4%) had the A411D change, and 1/13 (7.7%) had the A411T change. Based on the observed data, we hypothesize that the double mutation of N546H with T590S may positively affect viral replication and specific immune response. As shown in Table 3, the median hBoV load was significantly higher in samples of strains with the N546H and T590S changes than that in samples of wild type T590 strains, strains with only the T590S mutation, and strains with only the N546H mutation (p-values of 0.0078, 0.016 and 0.018, respectively). Finally, the two divergent strains (MI-267-Jan2014 and MI272-Jan2014) with unusual amino acid changes were observed with viral loads lower than 10^6^ DNA copies/mL. This could suggest that these mutations do not confer a replicative advantage in these virus strains.

**Table 3:** Presence of mutations in human bocavirus strains and relation of mutations with viral load.

Groups	No.	Viral load (DNA copies/mL		*P* value
		Median	Range	
A. Strains with T590	36	4.6 x 10^4^	3.5 x 10^2^–5.9 x 10^8^	
B. Strains with only T590S	61	4.5 x 10^4^	5.8 x 10^2^–7.5 x 10^9^	
C. Strains with only N546H	5	2.2 x 10^3^	3.5 x 10^2^–5.9 x 10^8^	
D. Strains with N546H+T590S	8	1.1 x 10^9^	1.6 x 10^4^–2.8 x 10^9^	
A *vs* B				0.31
A *vs* C				0.33
A *vs* D				**0.0078**
B *vs* C				0.17
B *vs* D				**0.016**
C *vs* D				**0.018**

### Clinical characteristics of hBoV1 infection

In Table 4, data regarding demographic, clinical and laboratory characteristics of children infected by hBoV-1 alone or co-infected with hBoV-1 and one or more other respiratory viruses are reported. Because a preliminary evaluation did not find any differences among subjects co-infected with EV/HRV, RSV or other viruses, all co-infections were considered together. As shown, children infected with only hBoV-1 had URTIs more frequently than those with a co-infection (37.0% vs 17.8%, respectively, p = 0.04). Moreover, a similar illness within the family in the 7 days since patient enrolment was significantly more common among co-infected children than among those with a single infection (48.9% vs 26.5%, respectively, p = 0.02). No other significant differences between the groups were observed.

**Table 4:** Comparisons between subjects positive for human bocavirus with or without co-infection(s), according to demographic, clinical and laboratory variables.^a^

Characteristic	Without co-infection(s) N = 57	With co-infection(s) N = 48	P value for comparison
	n/N (%)	n/N (%)	
**Demographic and clinical presentation**			
Males (%)	28/54 (51.8)	30/48 (62.5)	0.28
Mean age ± SD, yrs	2.30 ± 2.24	1.63 ± 1.31	0.06
Presence of fever” (%)	47/48 (97.9)	43/45 (95.6)	0.61
High-grade fever° (%)	24/48 (50.0)	25/45 (55.6)	0.59
Respiratory rate, breaths/min	34.7 ± 8.0	37.7 ± 10.3	0.66
SpO_2_ in room air, mean % ± SD	98.0 ± 1.7	96.8 ± 2.4	0.06
Clinical findings			
Cough	41/49 (83.7)	39/47 (83.0)	0.93
Rhonchi	2/49 (4.1)	4/47 (8.5)	0.43
Rales	15/49 (30.6)	15/47 (31.9)	0.89
Wheezes	7/49 (14.3)	9/47 (19.1)	0.52
Upper respiratory tract infection	17/46 (37.0)	8/45 (17.8)	**0.04**
Lower respiratory tract infection	25/55 (45.4)	24/46 (52.2)	0.50
**Clinical outcome**			
Hospitalisation rate, no.(%)	7/49 (14.3)	10/47 (21.3)	0.37
Duration of hospitalisation, mean days ± SD	7.4 ± 2.6	5.9 ± 2.2	0.35
Drug use, no. (%)			
Antibiotics	38/49 (77.5)	40/47 (85.1)	0.34
Antipyretics	42/49 (85.7)	39/47 (83.0)	0.71
Aerosol therapy	29/49 (59.2)	32/47 (68.1)	0.36
Absence from community, mean days ± SD	7.9 ± 5.0	9.2 ± 7.0	0.84
Similar illness within the family	13/49 (26.5)	23/47 (48.9)	**0.02**
**Laboratory data**			
White blood cell count (cells/μL)	15,319 ± 6,805	11,889 ± 3,680	0.27
Neutrophils, %	49.2 ± 28.4	38.5 ± 24.7	0.42
Lymphocytes, %^b^	26.3 ± 17.4	31.6 ± 4.6	0.99
Monocytes, %^b^	9.4 ± 4.6	11.2 ± 0.4	0.46
Basophils, %^b^	0.4 ± 0.3	0.2 ± 0.0	0.65
Eosinophils, %^b^	0.7 ± 0.6	0.6 ± 0.8	0.88
CRP, μg/dL	6.2 ± 15.4	1.4 ± 2.2	0.32

CRP: C reactive protein; SD: standard deviation; SpO_2_: peripheral oxygen saturation.”38.0°C or more any time during the illness (before or at enrolment, or during follow-up); **°**39.0°C or more any time during the illness (before or at enrolment, or during follow-up).

^a^Data were extracted from datasets of different studies that collected different information, therefore the denominators vary across characteristics.

^b^Information available for 9 subjects only (7 without co-infection and 2 with co-infection).

Table 5 shows the demographic, clinical, and laboratory characteristics of the enrolled subjects according to the hBoV load. Subjects with low and high viral loads were quite similar. The only significant differences were found in the duration of hospitalization, which was longer among children with a high viral load than among those with a low viral load (8.0 ±2.2 days vs 5.0 ±1.5 days, respectively, p = 0.03), and in the use of aerosol therapy, which was more frequent among children with a high viral load than among those with a low viral load (77.1% vs 55.7%, respectively, p = 0.04). Moreover, mutations leading to a high or low viral load were not associated with atypical clinical characteristics.

**Table 5:** Comparison between subjects with low and high viral human bocavirus loads, according to demographic, clinical and laboratory variables.^a^

Characteristic	Low viral load (≤10^6^ hBoV DNA copies/mL) N = 67	High viral load (>10^6^ hBoV DNA copies/mL) N = 38	P value for comparison
	n/N (%)	n/N (%)	
**Demographic and clinical presentation**			
Males (%)	40/64 (62.5)	18/38 (47.4)	0.14
Mean age ± SD, yrs	2.00 ± 2.04	1.98 ± 1.66	0.55
Presence of fever” (%)	58/58 (100.0)	32/35 (91.4)	0.05
High-grade fever° (%)	31/58 (53.5)	18/35 (51.4)	0.85
Respiratory rate, breaths/min	36.8 ± 8.6	36.6 ± 11.1	0.73
SpO_2_ in room air, mean % ± SD	97.3 ± 2.3	97.0 ± 2.1	0.49
Clinical findings			
Cough	48/61 (78.7)	32/35 (91.4)	0.11
Rhonchi	4/61 (6.6)	2/35 (5.7)	0.99
Rales	19/61 (31.1)	11/35 (31.4)	0.98
Wheezes	11/61 (18.0)	5/35 (14.3)	0.64
Upper respiratory tract infection	18/57 (31.6)	7/34 (20.6)	0.26
Lower respiratory tract infection	32/64 (50.0)	17/37 (46.0)	0.69
**Clinical outcome**			
Hospitalisation rate, no.(%)	10/61 (16.4)	7/35 (20.0)	0.66
Duration of hospitalisation, mean days ± SD	5.0 ± 1.5	8.0 ± 2.2	**0.03**
Drug use, no. (%)			
Antibiotics	51/61 (83.6)	27/35 (77.1)	0.43
Antipyretics	52/61 (85.2)	29/35 (82.9)	0.76
Aerosol therapy	34/61 (55.7)	27/35 (77.1)	**0.04**
Absence from community, mean days ± SD	8.6 ± 6.3	8.2 ± 5.5	0.88
Similar illness within the family	22/61 (36.1)	14/35 (40.0)	0.70
**Laboratory data**			
White blood cell count (cells/μL)	13,191 ± 6,847	14,727 ± 4,337	0.28
Neutrophils, %	41.9 ± 24.2	50.2 ± 32.1	0.52
Lymphocytes, %^b^	31.7 ± 13.1	19.1 ± 19.0	0.39
Monocytes, %^b^	11.7 ± 2.7	5.9 ± 3.6	0.09
Basophils, %^b^	0.4 ± 0.3	0.3 ± 0.4	0.90
Eosinophils, %^b^	0.7 ± 0.7	0.6 ± 0.5	0.99
CRP, μg/dL	5.5 ± 14.6	1.6 ± 1.8	0.94

CRP: C reactive protein; SD: standard deviation; SpO_2_: peripheral oxygen saturation.”38.0°C or more any time during the illness (before or at enrolment, or during follow-up); **°**39.0°C or more any time during the illness (before or at enrolment, or during follow-up).

^a^Data were extracted from datasets of different studies that collected different information, therefore the denominators vary across characteristics

^b^Information available for 9 subjects only (6 with low and 3 with high viral load).

## Discussion

This study shows that in Italy during the winter periods 2009–2010, 2011–2012, and 2013–2014, the incidence of hBoV infection among children with respiratory disease was relatively low, limited to approximately 5% of cases, and did not significantly vary from year to year.

The phylogenetic analysis showed that all of the strains detected in this study belonged to hBoV genotype 1 and were closely related to the prototype strain identified by Allander et al. [1]. This was expected because this genotype is the most common among hBoVs associated with respiratory infections [1].

Most of the patients in whom hBoV1 was identified were younger than 3 years of age, further highlighting that younger children are the individuals most frequently infected by this viral agent [1]. Serological studies have shown evidence that the number of subjects positive for anti-hBoV1 antibodies continuously increases with increasing age group from the ages of 6 months to 6 years, and by the age of 2 years approximately 80% of children have been infected with hBoV1 [16,17]. More than 50% of the children infected by hBoV1 in this study were co-infected with at least one other respiratory virus. Moreover, co-infected patients had LRTI more frequently than those infected by hBoV alone. These findings are not surprising because simultaneous detection of hBoV1 and other viruses in children with respiratory disease and greater severity in co-infected cases have been already reported in studies in which it was also demonstrated that hBoV1 can frequently be identified in the respiratory secretions of asymptomatic subjects [18–23]. Recently, it has been reported that hBoV1 can be shed for several days or months after a previous infection [24], which could explain the simultaneous identification of hBoV1 and other respiratory viruses, the frequency of asymptomatic infections and the generally greater severity of infections in co-infected individuals compared to those with hBoV1 alone. In most of the co-infected cases, detection of hBoV in the respiratory secretions with the new sensitive molecular methods able to identify very low viral loads might be a consequence of a previous clinically resolved disease, and a virus other than hBoV was therefore the real cause of the disease. However, reports of severe clinical manifestations in patients infected with only hBoV have been published [25], highlighting that the assessment of the real importance of hBoV infection in a single patient remains very difficult. Studies on children with severe pneumonia, acute wheezing, asthma and/or bronchiolitis suggested that hBoV1 is able to infect the lower airways down to the bronchioles [26–32]. Moreover, hBoV1 has been found as the only infectious agent in adult lung transplant recipients with severe LRTI, whereas it was not detected in respiratory secretions of asymptomatic transplanted subjects [33]. This would indicate that hBoV1 is not always a bystander or the cause of mild respiratory problems but rather a real, although relatively rare, causative agent of severe disease in both children and adults, particularly when they are immunocompromised. On the other hand, hBoV can cause serious neurological infections [34] and contribute to chronic disease in adult patients mainly because it can persist after childhood infection and reactivate [35].

Evaluation of the viral load has been considered a possible method to define when this virus is the real cause of a respiratory disease and when it is only a secondary infection. Unfortunately, this approach has had no success because although some studies have shown evidence for a strict correlation between high viral load and severe LRTI in children with a single hBoV infection [36–38], others, including the present study, did not show a clear relationship between these two variables [39].

However, the evolution of virulence appears to involve a variety of mechanisms in different viral systems, including mutations in regulatory regions and viral adaptation for utilization of alternative or expanded repertoires of cellular receptors. An alternative hypothesis to evaluate the importance of hBoV1 concerns the correlation between viral load levels and the presence of specific mutations. However, mutations associated with increased or reduced replication are rarely reported for hBoV. Recently, Hao et al. have reported that few nucleotide changes were correlated with a lower viral load [40]. In the present study, a double mutant (N546H and T590S) was observed in samples with a significantly higher viral load. However, further phenotypic validation studies are required to draw major conclusions regarding the impact of these mutations on viral replication. Furthermore, as reported by Qu et al. [41], it seems that nucleotide changes in the VP1U region could affect the replication efficiency of hBoV. Likewise, in our strains all the amino acids of the catalytic site were conserved, and no mutations that affect sPLA_2_ activity were identified.

In agreement with others [42], the phylogenetic analysis of this study confirmed the very low degree of variability in the hBoV genomic region encoding proteins that are exposed to the virus surface and are therefore under immunologic pressure. Only 9% (62 codons) of amino acids were found to have at least one change in the VP1/VP2 gene, a finding not substantially different from that reported by Hao et al. in a different geographic area [40]. In our study, several amino acid changes were observed in strains circulating in almost all the respiratory seasons. This finding provides evidence that the selection of those variants best adapted to each particular environment might select for variants with an evolutionary advantage. Seven of these mutations were located in a genomic region (i.e., VP1U) previously reported to be involved in the mechanisms of virus replication. For instance, the VP1U amino acid variations R17K and L40S have been previously reported [43], whereas the remaining variations have not yet been described. Interestingly, two hBoV strains identified in respiratory samples collected in January 2014 had unusual amino acid sequences in a somewhat conserved genomic region. The reason for this genetic diversity is still undefined. However, as described for hBoV, other parvoviruses [44], and enteroviruses, a series of α-helices and β-barrels in the VP2 protein were intercalated by an external loop, in which the majority of the genetic variability accumulated. Nevertheless, these two divergent strains were found in samples with low viral loads, and we might hypothesize a loss of replication advantage for these strains.

In the present study, the dN/dS ratios for all pairwise comparisons were <1, which is in line with previous results showing that positive selection was extremely limited in parvoviruses [45]. In fact, selective pressure analyses have identified 3 codons under positive selection. In a previous paper, a different codon (L40) was identified as being under positive selection [46]. Nevertheless, the great majority of codons were under negative or neutral selection, which has also been confirmed by others [47]. This finding suggests that only a few amino acids of the VP1/VP2 proteins present on the surface of the virion are potentially subjected to a strong selective pressure by the host immune response.

In conclusion, this study confirms that hBoV is less common than other respiratory viruses but that the frequency of its detection in children with respiratory disease is in time stable. It was detected with a prevalence of about 5% in several consecutive seasons and no unusual clustering was observed among identified strains, with strains circulating in 2009 being closely related to those circulating in 2014. Moreover, only a minority of virus sites were found to be under positive selective pressure, and all the strains detected in respiratory tract infections of this Italian study belonged to genotype 1. From a clinical point of view, this study highlights that in otherwise healthy children, hBoV1 seems to have relatively low clinical relevance, because patients infected with hBoV alone mainly suffered from an URTI. The viral load was not associated with clinical characteristics of the infection, and viral mutations, despite affecting viral replication, did not affect the conditions or severity of the clinical presentation. Further studies are needed to clarify the clinical relevance of hBoV in children, particularly in those at risk for severe chronic underlying disease, and to evaluate the role of viral modification in conditioning the degree of viral virulence and the specific immune response.
